# Identifying the mechanism of polysaccharopeptide against breast cancer based on network pharmacology and experimental verification

**DOI:** 10.1186/s12885-024-12494-1

**Published:** 2024-06-13

**Authors:** Cuixiang Xu, Lijun Sun, Huxia Wang, Jingying Sun, Yangmeng Feng, Xingguang Wang, Zhangjun Song

**Affiliations:** 1 Shaanxi Provincial Key Laboratory of Infection and Immune Diseases, Shaanxi Provincial People’s Hospital, Xi’an, 710068 Shaanxi China; 2 Shaanxi Province Research Center of Cell Immunological Engineering and Technology, Xi’an, 710068 Shaanxi China; 3 Department of Breast Disease Center, Shaanxi Provincial Cancer Hospital, Xi’an, 710065 Shaanxi China; 4 Department of Surgical Oncology, Shaanxi Provincial People’s Hospital, 256 Youyi Road, Xi’an, 710068 Shaanxi China

**Keywords:** Breast cancer, Polysaccharopeptide, Network pharmacology, Molecular docking, Experimental validation

## Abstract

Polysaccharopeptide (PSP) is a potential active component in traditional Chinese medicine because of its anticancer effects on a variety of cancer cells and as immune enhancers of the immune system. Previous studies on the role of PSP in breast cancer have been limited, and the mechanism has not been clarified. This study is based on network pharmacology and molecular docking technology to predict the possible target of PSP treatment of breast cancer, and use experiments to verify the effect and mechanism of PSP on breast cancer. In this study, 287 PSP targets were obtained using SwissTargetPrediction database and PharmMapper database, and 183 breast cancer targets were obtained using DisGenNET database. By intersections of PSP targets and breast cancer targets, a total of 10 intersections were obtained. GO functional enrichment, KEGG pathway enrichment and molecular docking of these 10 target genes were performed to obtain the potential targets of PSP on breast cancer. In vitro experiments, we found that PSP significantly inhibited the proliferation and induced apoptosis of breast cancer cell lines MDA-MB-231, SUM-159 and MCF-7. Western Blot results showed that PSP could down-regulate the expression of p-JAK2 and p-STAT3 proteins. Similarly, the results of in vivo experiments showed that PSP can directly inhibit the tumor of MDA-MB-231 tumor-bearing mice, and the mechanism of action is mainly to inhibit the JAK2-STAT3 pathway. The above results were consistent with the results of network pharmacology, which provides a scientific basis for the clinical application of PSP in breast cancer patients.

## Introduction

Breast cancer has become the most important malignancy threatening the health of women all over the world. In recent years, chemotherapy, targeted therapy and other methods have significantly improved the survival rate of breast cancer, but there are still problems in the treatment of breast cancer such as large side effects and easy drug resistance [1]. Therefore, it is necessary to find new drugs with obvious effect, clear mechanism, safety and non-toxic.

Coriolus versicolor (CV), a quality medicine that promotes health and longevity, has been used in traditional Chinese medicine for more than 2,000 years [2]. Polysaccharopeptide (PSP) is the most active biological component of CV and can be obtained from its mycelium or fermentation broth. PSP has anti-cancer effects on a variety of cancer cells, and they can also act as immune enhancers of the immune system [3]. Chow et al. found that PSP can up-regulate P21 gene and down-regulate Cyclin D1 to inhibit cell proliferation in breast cancer cell line MDA-MB-231 [4]. Tsang et al. found that PSP could delay the progression of non-small cell lung cancer [5]. Sekhon et al. found that PSP can significantly increase CD14^+^/CD16^−^ type macrophages, which can better improve human immunity and eliminate tumor cells [6]. In addition, the combination of PSP and gamma-tocotrienol can enhance the anti-tumor effect of gamma-tocotrienol and reduce its side effects [7].

At present, PSP is mostly used in clinical adjuvant therapy for chemotherapy and radiotherapy patients, but its effect and mechanism on breast cancer are still unclear. Network pharmacology is an emerging discipline based on the organic combination of medicine, computer science and pharmacology [8]. Our study applied network pharmacology to further explore the anti-tumor mechanism of PSP and provide a theoretical basis for the application of PSP in breast cancer treatment.

## Materials and methods

### Materials

Three breast cancer cell lines (MDA-MB-231, SUM-159 and MCF-7) and one human normal mammary epithelial cell line (MCF-10 A) were purchased from Procell Life Technology, Wuhan, China. DMEM and DMEM/F12 medium were purchased from Gibco (Carlsbad, CA, USA), fetal bovine serum was purchased from Tianhang Biotechnology, Zhejiang, China. The CCK8 and TUNEL staining kits were purchased from Elabscience Biotechnology, Wuhan, China. The Annexin V-FITC apoptosis detection kits was purchased from TransGen Biotech, Beijing, China. The rabbit anti-human Phospho-STAT3 antibody, rabbit anti-human Phospho-JAK2 antibody, rabbit anti-human STAT3 antibody, rabbit anti-human JAK2 antibody were purchased from Cell Signaling Technology, Boston, USA. The rabbit anti-human β-actin antibody, horseradish peroxidase (HRP)-labeled goat anti-rabbit IgG and HRP-labeled goat anti-mouse IgG were purchased from CWbio, Beijing, China. The rabbit anti-mouse Ki-67 and D-Luciferin were purchased from Abcam, Cambridge, UK. PSP powder (batch number:32,025,831) was purchased from Shenhua Pharmaceutical Co., Ltd, Jiangsu, China.

### Data collection of potential targets

Using Chem3D software to draw PSP molecular formula, then submit the molecular structure to the SwissTargetPrediction database (http://www.swisstargetprediction.ch/) and PharmMapper database (http://www.lilab-ecust.cn/pharmmapper/) to get the targets of the PSP. Using DisGeNET database (https://www.disgenet.org/) with “Breast Cancer” key words to search out the targets of Breast Cancer. Venn Diagram was drawn using VennDiagram plot.

### PPI network construction

In order to clarify the interactions among potential targets for PSP treatment of breast cancer and obtain the drug-disease interaction network diagram, we imported the targets into the STRING database (https://string-db.org) to obtain the protein-protein interaction (PPI) network diagram.

### GO and KEGG pathway enrichment analysis

In order to further clarify the role of PSP in the treatment of breast cancer, GO and KEGG enrichment analysis of target genes was performed using Rstudio software, and the biological processes, molecular functions and signaling pathways involved in the main targets of PSP were obtained.

### Molecular docking

The PDB database was used to download the crystal structure of target proteins. The PubChem database (https://pubchem.ncbi.nlm.nih.gov/) was used to download the 3D structure of PSP. AutoDockTools 1.5.6 software was used for molecular docking and the result was processed by Pymol software.

### Cell cultures

Three human breast cancer cell lines, including SUM-159, MCF-7 and MDA-MB-231 were maintained in DMEM medium supplemented with 10% FBS and 1% antibiotics. Human normal mammary epithelial cell line MCF-10 A was maintained in DMEM/F12 medium supplemented with 10% FBS and 1% antibiotics. The cells were incubated at 37 ℃ with 5% CO_2_.

### Cell viability assays

The proliferation ability of the cells was detected by CCK-8 kit. Cell suspension was prepared and inoculated into 96-well plates with 2000 cells per well. Different concentrations of PSP were added to the cells and samples were collected after 48 h and 72 h. 10 µl CCK-8 solution was added to each well and incubated for 2 h. The absorbance at 450 nm was measured using a microplate reader (HEALES, MB-580).

### Colony formation assays

Cell suspension was prepared, 200 cells were inoculated in each plate, then the cells were treated with PSP and incubated in 5% CO_2_ at 37℃ for 7–10 days. The culture was terminated and the supernatant of the cells was discarded, and the cells were washed twice with PBS, fixed for 15 min, and then stained with crystal violet. The plates were photographed, cell clones were counted and the rate of clone formation was calculated.

### Flow cytometry assay

The Annexin V-FITC apoptosis detection kit was used for analysis according to the manufacturer’s instructions. Briefly, cells were digested with EDTA-free pancreatase, centrifuged at 4℃ for 5 min and collected. Then the cells were washed twice with pre-cooled PBS and re-suspended with 100 µl pre-cooled Binding Buffer. 5 µl Annexin V-FITC and 5 µl propidium iodide were added respectively, and the reaction was in a darkroom at 37℃ for 15 min. Finally, 400 µl pre-cooled Binding Buffer was added and the samples were detected by Flow Cytometry (Agilent).

### Tunel staining

The apoptosis of cells was evaluated via the Tunel Apoptosis Assay Kit. The cells were fixed at room temperature for 15 min, washed with PBS for 3 times, then incubated with permeable solution at room temperature for 10 min. Each sample was added with 100 µl Tunel detection solution, reacted at 37℃ for 30 min, and washed with PBS for 3 times. DAPI working solution was added and incubated for 5 min at room temperature in a darkroom to stain the nucleus. The samples were observed and photographed under fluorescence microscope (Olympus Corporation).

### Western blotting analysis

Total proteins were extracted from cell samples and tumor tissue samples. The proteins were separated by SDA-PAGE gels, transferred to NC membranes, sealed with 5% skim milk at room temperature for 2 h, and then incubated with antibodies including p-JAK2 (1:500), p-STAT3 (1:1000), JAK2 (1:500), STAT3 (1:1000), β-actin (1: 1000) at 4℃ overnight. The membranes were cleaned with PBST for 3 times and incubated with HRP-conjugated secondary antibodies for 40 min at room temperature. Finally, the blots were detected using a gel imaging system (Alpha Innotech).

### Tumor model in vivo

Female Balb/c mice (6–8 weeks old) were obtained from the Experimental Animal Center of Xi’an Jiaotong University. The experiment was approved by the Biomedical Ethics Committee of Health Science Center of Xi’an Jiaotong University. MDA-MB-231 cells with stable luciferase expression were collected, and 1 × 10^6^ cells were inoculated subcutaneously in each Balb/c mouse. When the tumor volume reached 100 mm^3^, the mice were randomly divided into negative control group, 400 mg/kg experimental group, 800 mg/kg experimental group and positive control group. The negative control group was gavaged with PBS everyday, the experimental group was gavaged with PSP everyday, and the positive control group was intraperitoneal injection of cisplatin (2 mg/kg), once every 3 days. The weight of the mice was measured every week, and the length (L) and width (W) of the tumor were measured with a caliper to calculate the volume of the tumor. Mice were imaged after 14 and 23 days of drug action. 15 mg/ml D-Luciferin potassium salt solution was injected intraperitoneally into mice, and live imaging was performed by AniView 600 animal imaging system (Boruteng Biotechnology). Thirty days later, the mice were anesthetized by inhalation of isoflurane, then euthanized by cervical dislocation. The tumors, hearts, livers, spleens, lungs and kidneys of mice were dissected for corresponding analysis.

### Hematoxylin-eosin staining

The tissues were fixed in 4% paraformaldehyde for 24 h, dehydrated, and embedded in paraffin. Sections 4 μm thick were stained with hematoxylin and eosin, respectively. Images were observed and photographed under the BX41 fluorescence microscope (Olympus Corporation).

### Immunohistochemistry

Paraffin sections were dewaxed and incubated at room temperature with 3% H_2_O_2_ for 20 min to eliminate endogenous peroxidase. Wash three times with PBS for 5 min each time. After being blocked with 10% normal goat serum at room temperature for 20 min, the sections were incubated with primary antibodies at 4℃ overnight. HRP labeled secondary antibodies were added and incubated at 37℃ for 20 min. The sections were stained in hematoxylin for 3 min and observed under the BX41 fluorescence microscope (Olympus Corporation).

### Statistical analysis

Each experiment was independently performed three times, with data expressed as mean ± SD. Differences between groups were analyzed using one-way ANOVA. Differences of *p* < 0.05 were considered statistically significant.

## Result

### Anti-breast cancer targets of PSP analysis

**Fig. 1:**
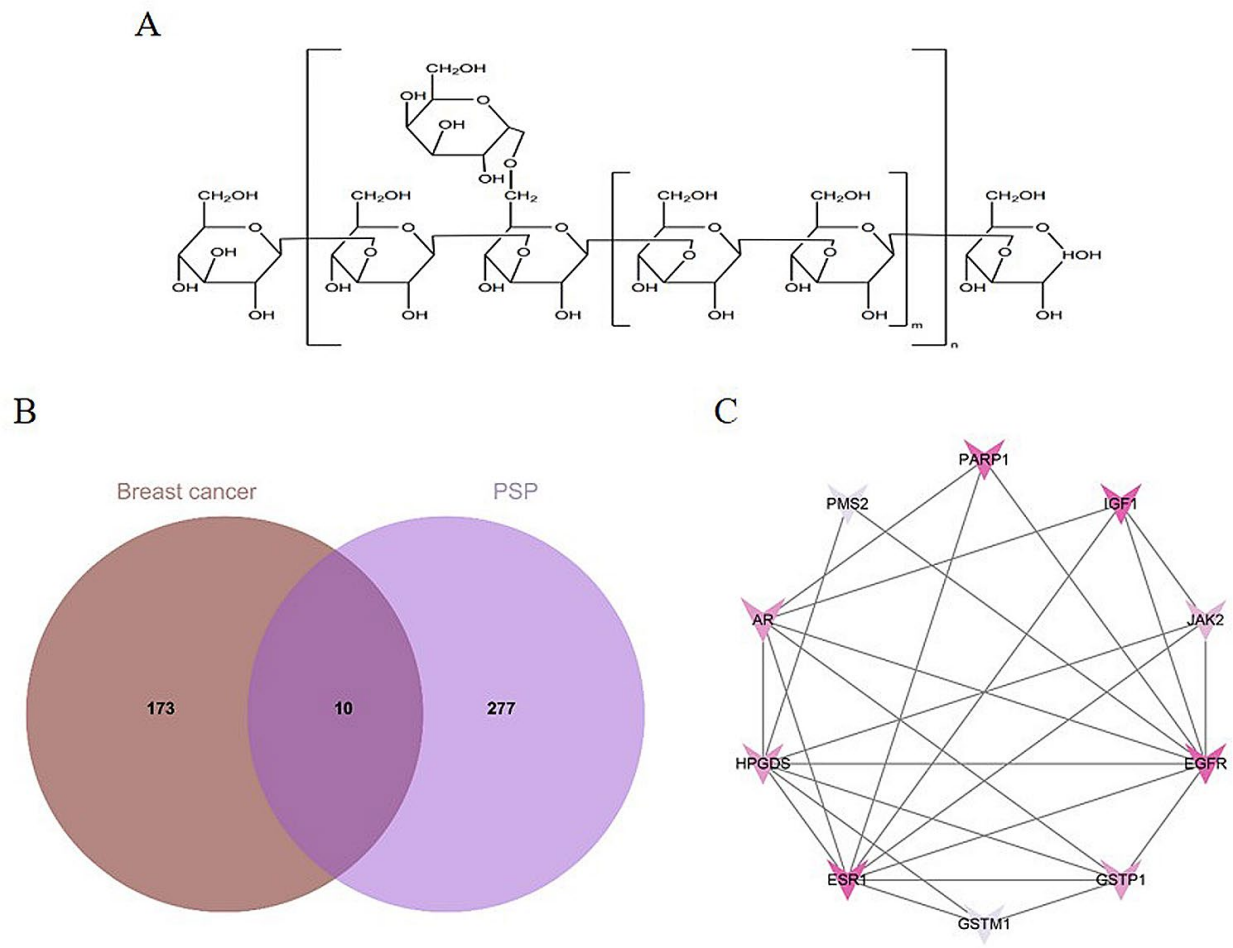
Network pharmacological research on the anti-breast cancer target of PSP. (**A**) Chemical structure of PSP. (**B**) Venn diagram of PSP-breast cancer interactive targets. (**C**) Protein-protein interaction (PPI) network of 10 PSP anti-breast cancer targets

PSP molecular formula is shown in Fig. 1A. A total of 287 PSP targets were obtained using SwissTargetPrediction database and PharmMapper database, and 183 breast cancer targets were obtained using DisGenNET database. By intersections of PSP targets and breast cancer targets, a total of 10 intersections were obtained, as shown in Fig. 1B of Venn Diagram. The protein-protein interaction (PPI) network of 10 selected intersection targets were obtained using the STRING database, and the results were shown in Fig. 1C. The darker the color, the stronger the interaction ability of the target with other targets.

### Biological function and pathway enrichment analyses

**Fig. 2:**
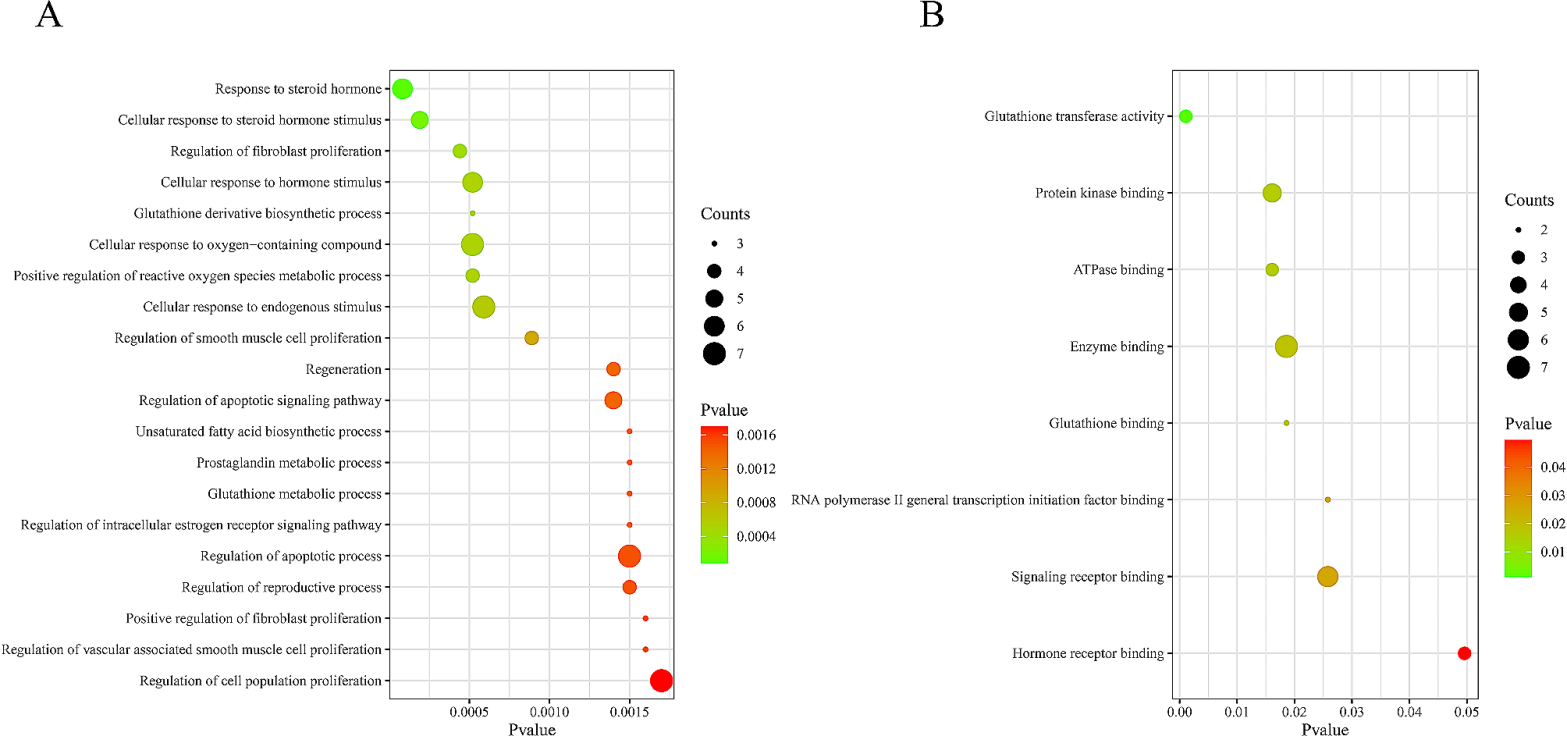
GO enrichment analysis for PSP anti-breast cancer targets. (**A**) Biological processes GO enrichment analysis. (**B**) Molecular function GO enrichment analysis

**Fig. 3:**
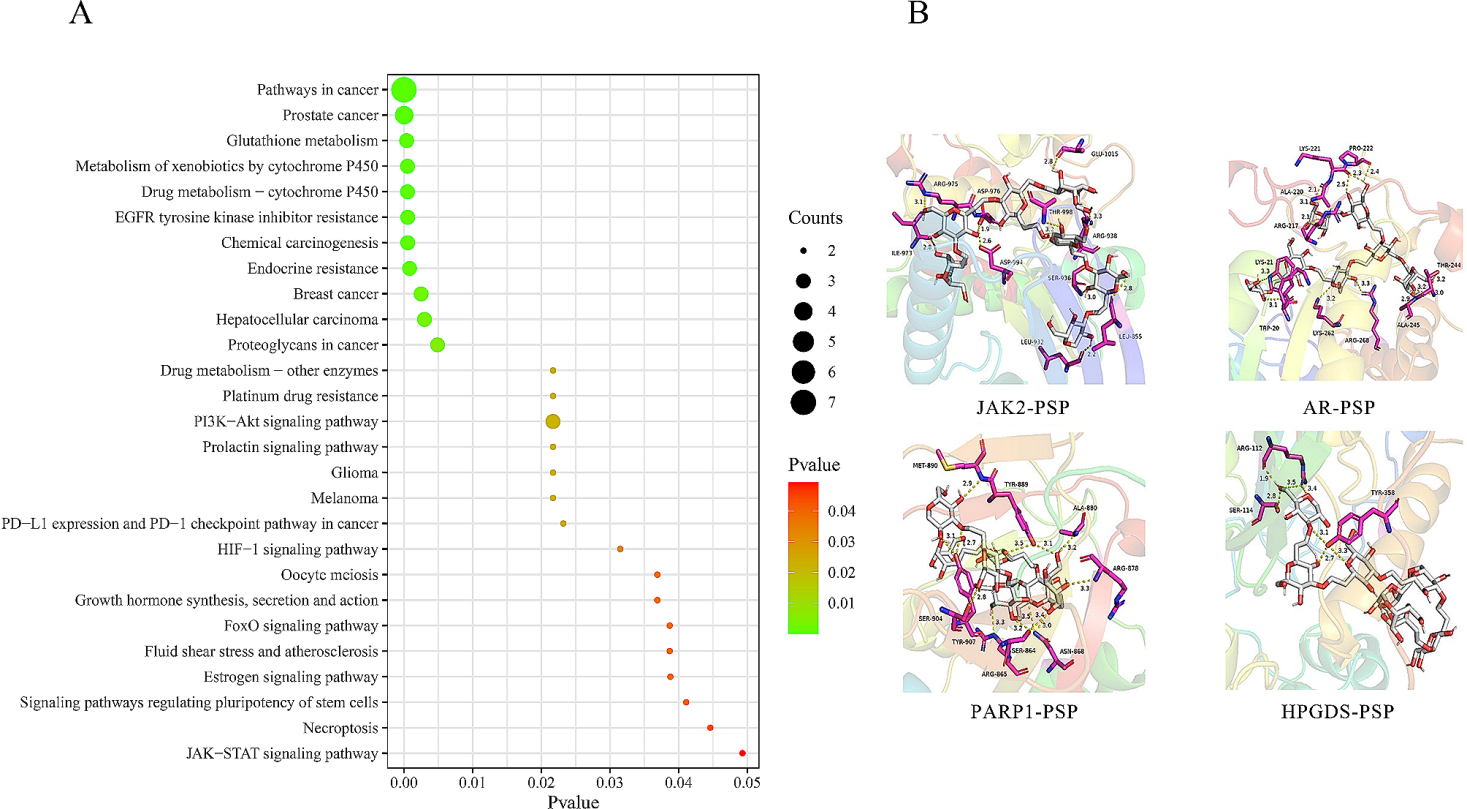
KEGG pathway analysis and molecular docking for PSP anti-breast cancer targets. (**A**) KEGG enrichment bubble diagram. (**B**) Molecular docking diagram of PSP and target proteins

GO and KEGG enrichment analysis of key genes in PSP treatment of breast cancer was performed by Rstudio software. 140 biological processes (BP) were obtained, which mainly involved the response to steroid hormones, regulation of fibroblast proliferation, cell response to oxygen compounds, regulation of apoptosis signaling pathways, etc. Sort according to the P-value and select the first 20 items to draw the bubble maps (Fig. 2A). 8 molecular functions (MF) were identified, involving glutathione transferase activity, protein kinase binding, adenosine triphosphatase binding, and signal receptor binding (Fig. 2B). A total of 27 key pathways were obtained by KEGG enrichment analysis, including pathways in cancer, prostate cancer, glutathione metabolism, breast cancer, PI3K-AKT, HIF-1, JAK-STAT and other signaling pathways (Fig. 3A).

### Molecular docking analysis

Since the KEGG pathway did not contain the target PMS2, it was excluded and the remaining 9 targets were used for molecular docking. The binding energy of targets and PSP were shown in Table 1. The higher the absolute value of binding energy, the stronger the binding ability of PSP to the targets. Four pairs of PSP-targets with binding energy less than − 6.5 kcal/mol were visualized. The results showed that JAK2, HPGDS, AR and PARP1 had good potential for targeted treatment of breast cancer (Fig. 3B).

**Table 1:** Results of molecular docking between PSP and the predicted targets

Targets	Affinity(kcal/mol)	PDB ID
JAK2	-8.4	3UGC
HPGDS	-7.2	1DGF
AR	-6.9	ZFZB
PARP1	-6.5	6NRH
ESR1	-6.4	7BAA
GSTP1	-6.1	1PKG
IGF1	-6.1	1XH7
EGFR	-5.9	6TFV
GSTM1	-5.4	1YZG

### PSP inhibits the proliferation of breast cancer cells

**Fig. 4:**
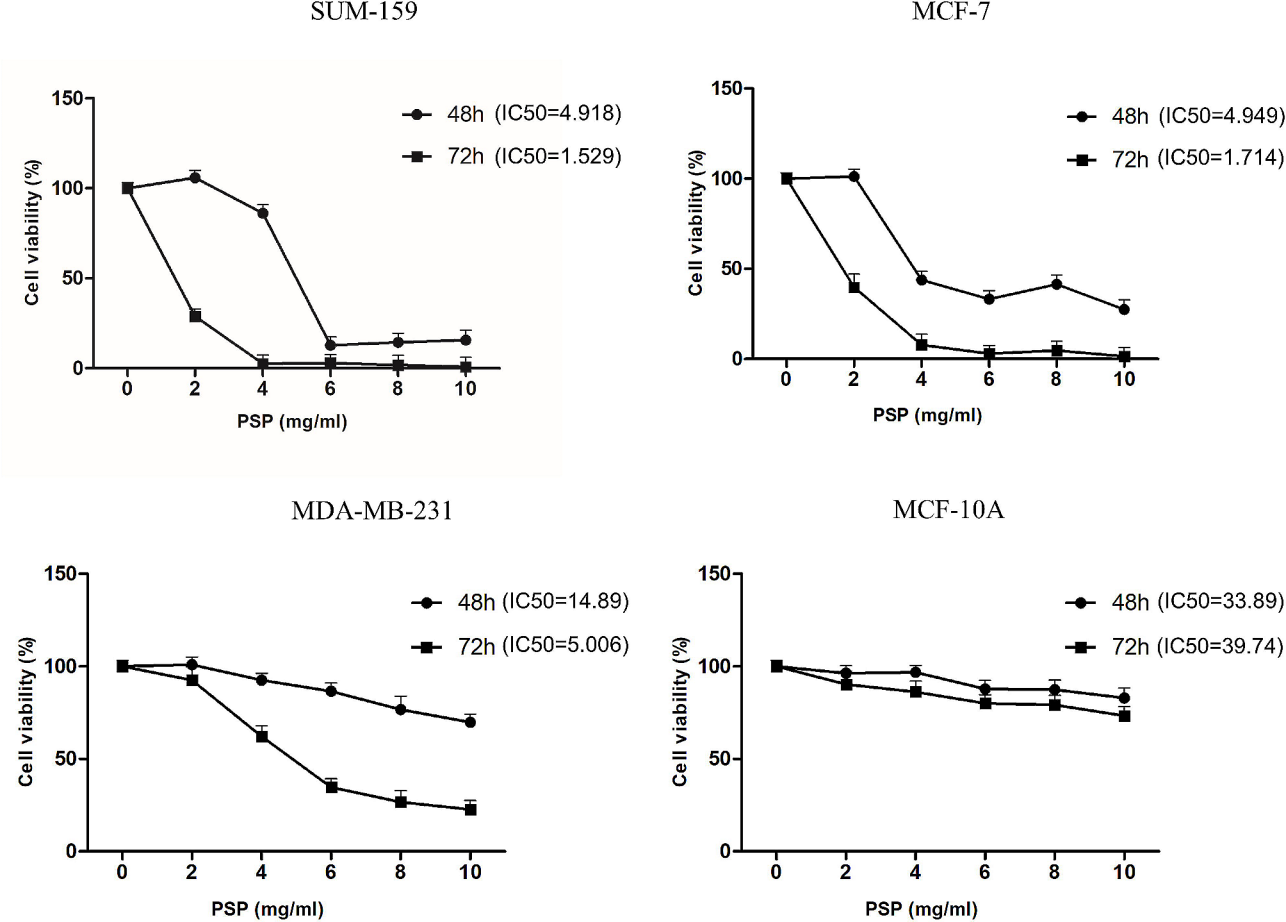
PSP inhibits the proliferation of breast cancer cells. The viability of MDA-MB-231, SUM-159, MCF-7 and MCF-10 A cells treated with different concentrations of PSP (0, 2, 4, 6, 8, 10 mg /ml) for 48 and 72 h was detected by CCK-8 assay

Three breast cancer cell lines (SUM-159, MCF-7 and MDA-MB-231) and one human normal mammary epithelial cell line (MCF-10 A) were used to examine the effect of PSP on cell proliferation. CCK-8 assay results showed that PSP inhibited breast cancer cell proliferation in a dose- and time-dependent manner, but PSP was less sensitive to normal breast cell line MCF-10 A than breast cancer cells (Fig. 4). The IC50 values of PSP for SUM-159, MCF-7, MDA-MB-231 and MCF-10 A cells after 72 h of treatment were 1.529 mg/ml, 1.714 mg/ml, 5.006 mg/ml and 39.74 mg/ml, respectively.

### PSP inhibits colony formation of breast cancer cells

**Fig. 5:**
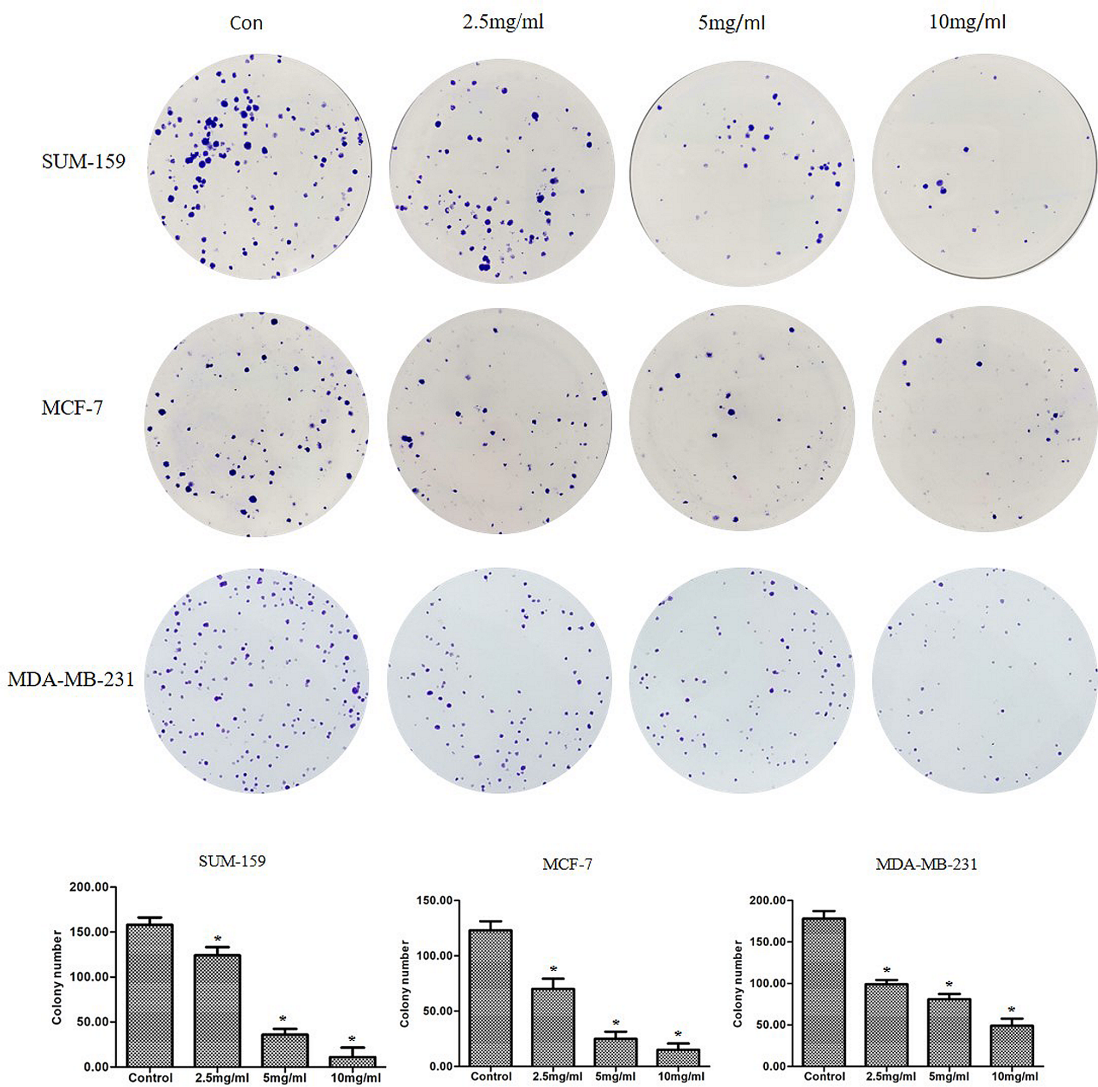
PSP inhibits colony formation of breast cancer cells. MDA-MB-231, SUM-159 and MCF-7 cells were treated with different concentrations of PSP for 15 days, cell clones were stained and counted. *P < 0.05

Breast cancer cell lines were treated with different concentrations of PSP (0, 2.5, 5 and 10 mg/ml) for 7–10 days, and colony formation experiments showed that the number and size of cell clones decreased with the increase of PSP concentration. The results showed that PSP inhibited the proliferation of SUM-159, MCF-7 and MDA-MB-231 breast cancer cells in a dose-dependent manner compared with non-treated groups (*P < 0.05*) (Fig. 5).

### PSP induced apoptosis of breast cancer cells

**Fig. 6:**
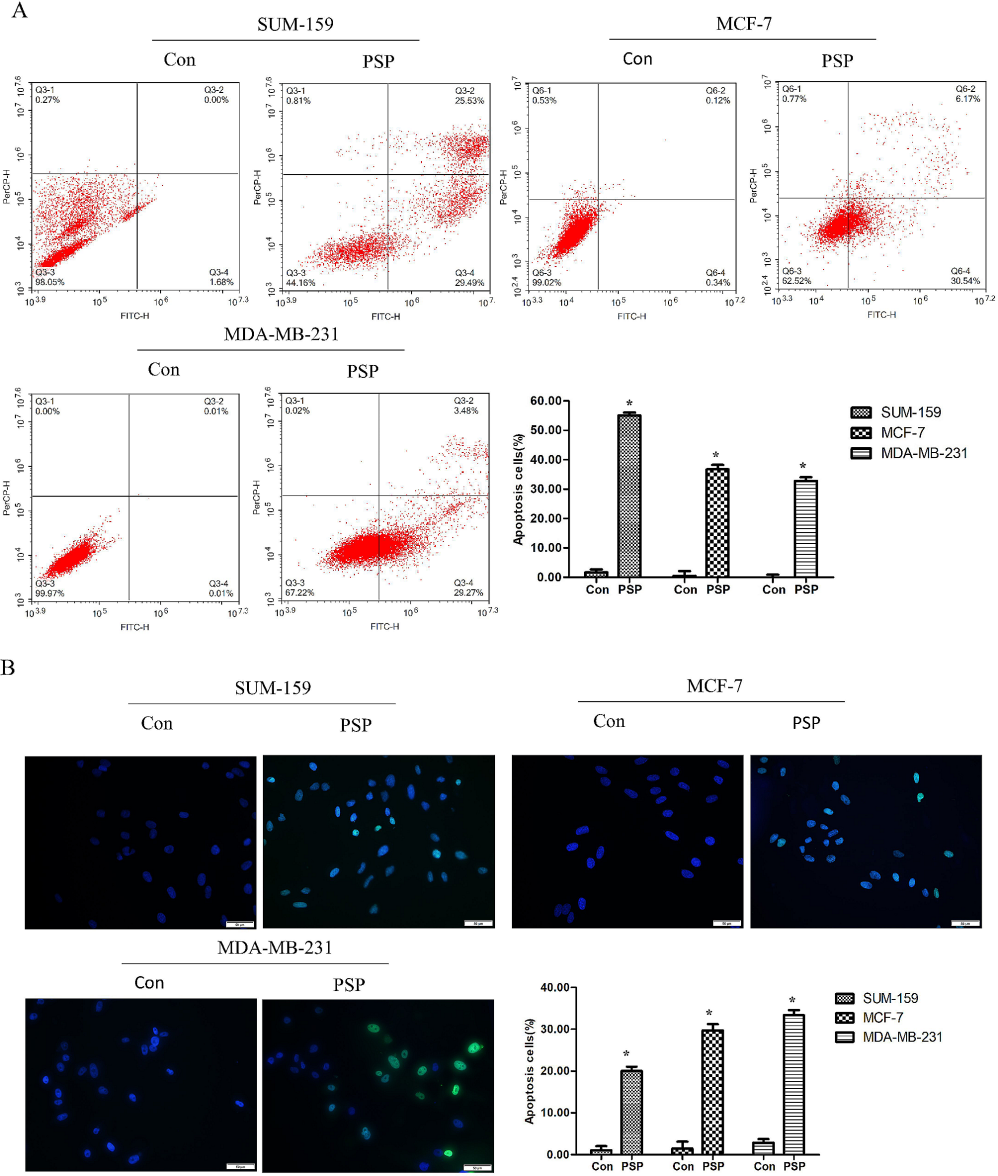
PSP induced apoptosis of breast cancer cells. (**A**) The effect of PSP on apoptosis of MDA-MB-231、SUM-159 and MCF-7 cells was determined by flow cytometry analysis. (**B**) The apoptosis-positive cells were stained with Tunel staining kit. *P < 0.05

Breast cancer cell lines were treated with 5 mg/ml PSP for 24 h, Tunel staining and flow cytometry were used to evaluate the effect of PSP on apoptosis of breast cancer cell lines. Flow cytometry results showed that compared with the control group, PSP treatment increased the apoptosis rate of SUM-159, MCF-7 and MDA-MB-231 cells (*P < 0.05*) (Fig. 6A). Similarly, Tunel staining showed that the apoptosis rate of the PSP treated group was significantly increased compared with the control group (*P < 0.05*) (Fig. 6B).

### Effect of PSP on JAK2/STAT3 signaling pathway in breast cancer cells

**Fig. 7:**
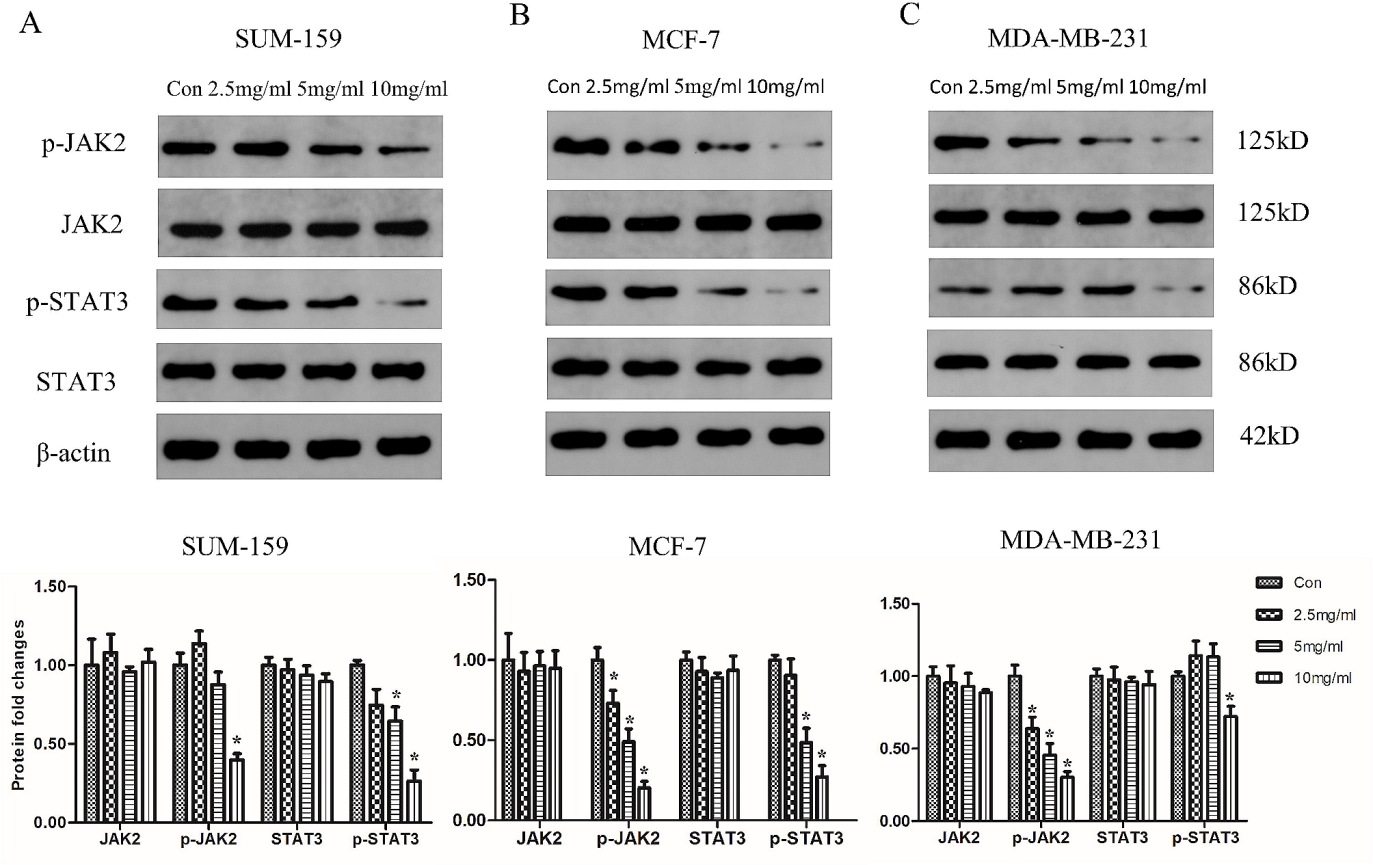
PSP inhibits the JAK2/STAT3 signaling pathway in breast cancer cells. (**A**) The expression levels of p-JAK2, JAK2, p-STAT3 and STAT3 protein in SUM-159 cells. (**B**) The expression levels of p-JAK2, JAK2, p-STAT3 and STAT3 protein in MCF-7 cells. (**C**) The expression levels of p-JAK2, JAK2, p-STAT3 and STAT3 protein in MDA-MB-231 cells. Lane-I-V represented cells with 0, 2.5, 5, 10 mg/ml PSP treatment group. *P < 0.05

Molecular docking results indicated that JAK2 was a potential target of PSP in breast cancer, and KEGG analysis showed that the JAK-STAT pathway was enriched. Therefore, western blot was used to detect the expression of JAK2, STAT3, p-JAK2 and p-STAT3 proteins after the cells were treated with different concentrations of PSP. The results showed that the expression levels of p-JAK2 and p-STAT3 in SUM-159, MCF-7 and MDA-MB-231 cells were significantly decreased after PSP treatment, and the decrease trend was concentration-dependent, while the expression levels of JAK2 and STAT3 were not significantly changed. These results suggest that PSP can inhibit the JAK2-STAT3 pathway, which is consistent with the results of bioinformatics analysis (Fig. 7A-C).

### PSP inhibits tumor growth in mouse models

**Fig. 8:**
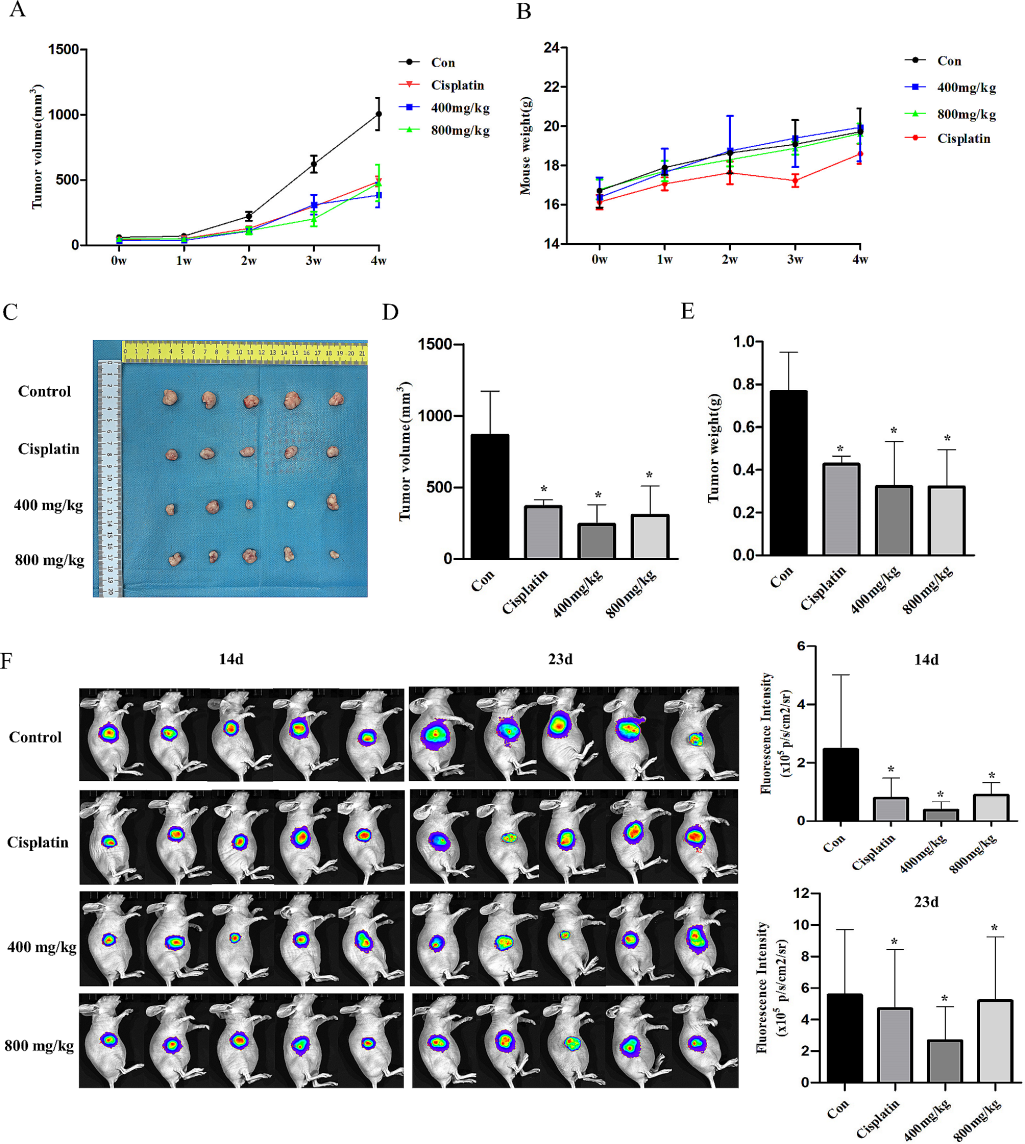
PSP inhibits tumor growth in vivo. (**A**) Tumor size was measured every week after implanting of MDA-MB-231 cells. (**B**) The body weight of mice was measured every week. (**C**) Picture of the tumor after the mice were euthanized. (**D**) Tumor volume was measured after 30 days treatment in the PBS, PSP (400 mg/kg), PSP (800 mg/kg) and cisplatin groups. (**E**) The weight of the tumor was measured after 30 days treatment in the PBS, PSP (400 mg/kg), PSP (800 mg/kg) and cisplatin groups. (**F**) Live images of mice tumor after 14 and 23 days in the PBS, PSP (400 mg/kg), PSP (800 mg/kg) and cisplatin groups. *P < 0.05

We used MB-MD-231 xenograft mouse models to investigate the effect of PSP on tumor growth in vivo. PSP was administered to MBA-MD-231 tumor-bearing mice by gavage from day 7 to day 36 after tumor implantation. The negative control group was gavaged with PBS, and the positive control group was intraperitoneal injected with cisplatin. Cisplatin is a first-line chemotherapy drug for breast cancer, so it was chosen as a positive control. The results showed that the PSP and cisplatin groups significantly delayed tumor growth in the mouse model compared to the negative control group, while the body weight of the mice did not change significantly (Fig. 8A and B). The size and weight of the tumors were measured after the mice were sacrificed on day 36. The PSP and cisplatin groups significantly reduced tumor size and weight compared to the control group (Fig. 8C-E). Live imaging of mouse tumors showed the same changes after 14 and 23 days of PSP treatment (*P < 0.05*) (Fig. 8F).

### Mechanism of PSP inhibiting tumor growth in vivo

**Fig. 9:**
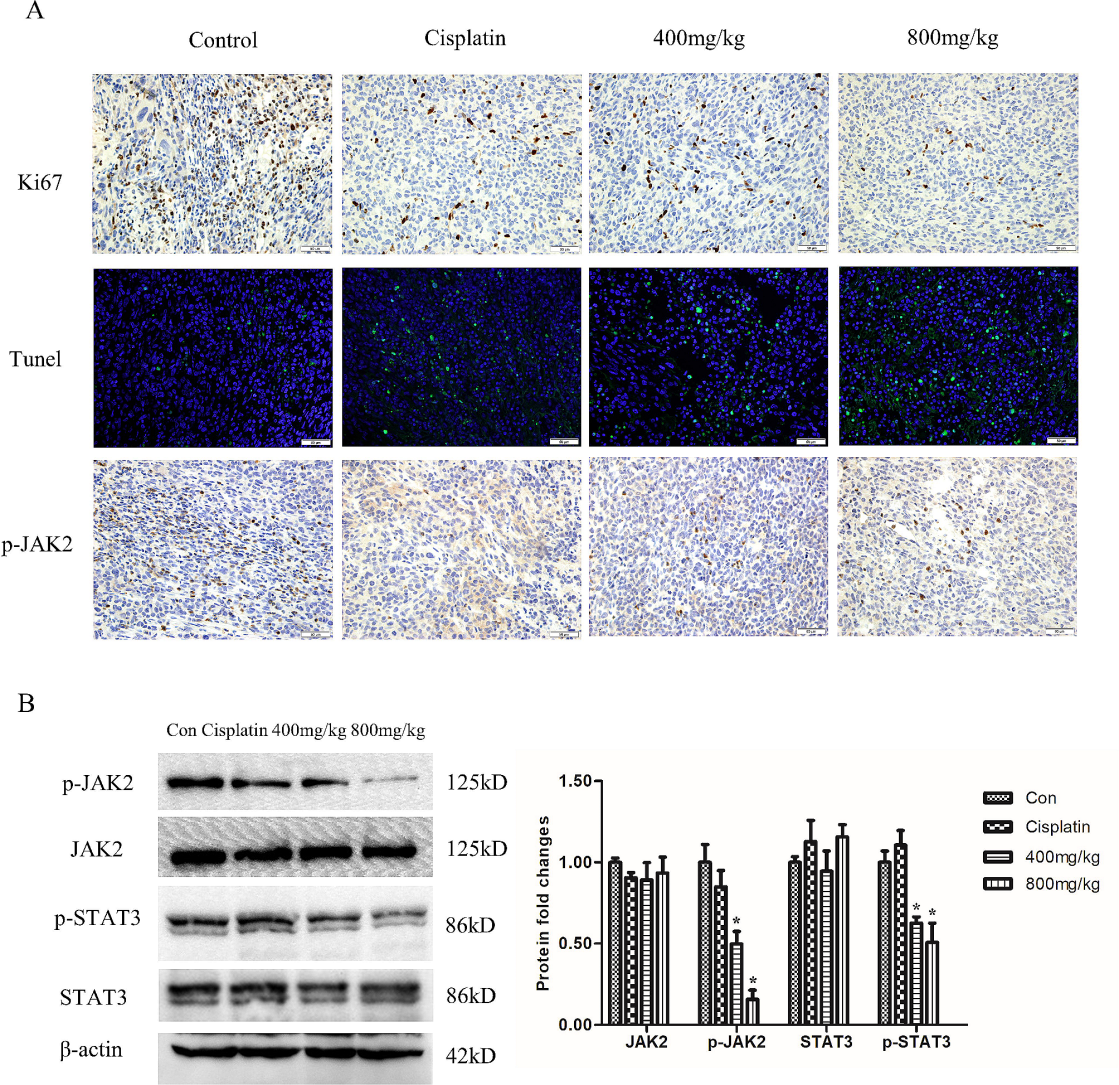
PSP inhibits tumor proliferation and induces apoptosis in vivo. (**A**) The expressions of Ki67 and p-JAK2 in tumor tissues were detected by immunohistochemistry. Apoptotic cells in tumor tissues were stained with Tunel kit. (**B**) The expressions of p-JAK2, JAK2, p-STAT3 and STAT3 protein in tumor tissues were analyzed by Western blot. *P < 0.05

**Fig. 10:**
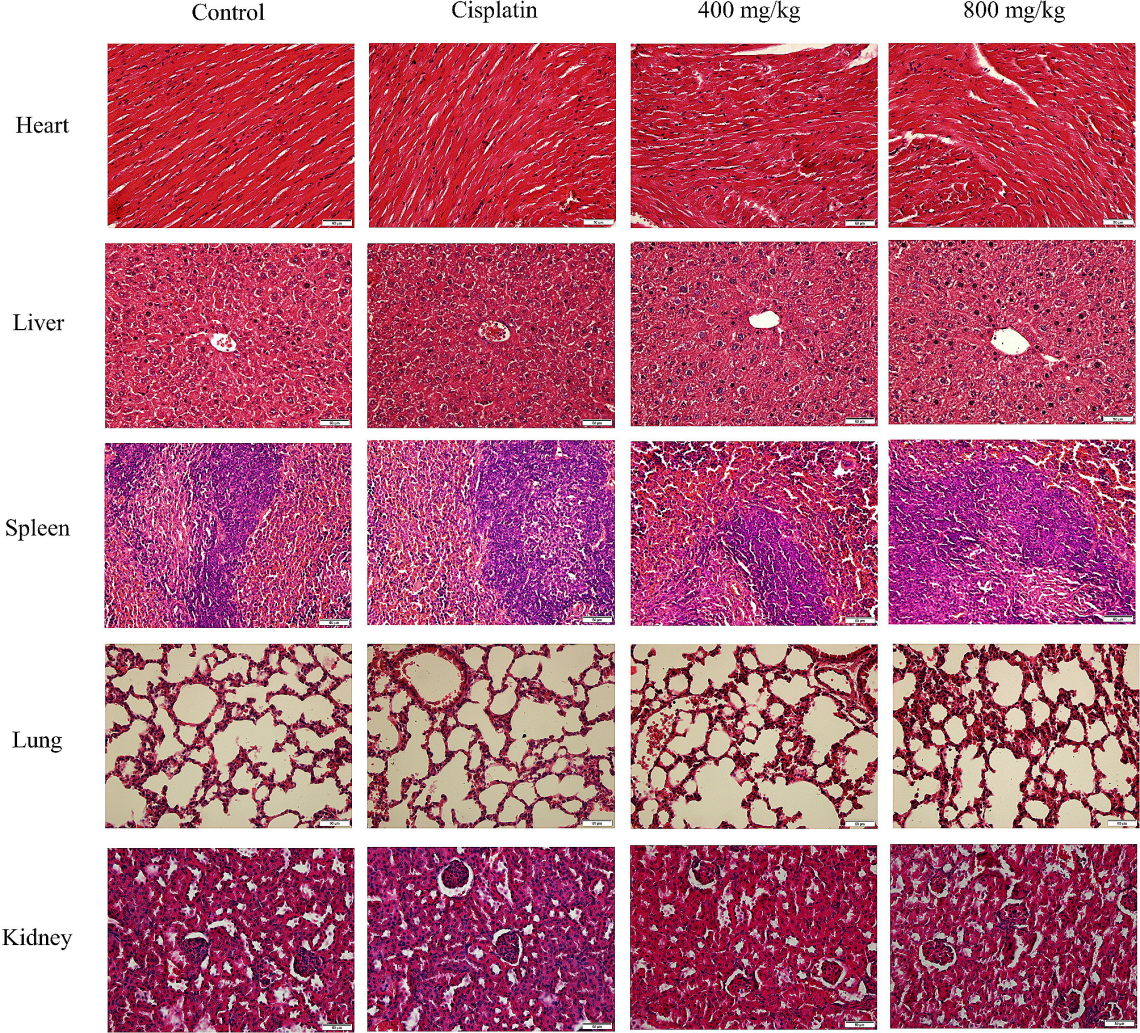
PSP had no side effects in major organs in vivo. HE staining was performed on tissue sections of heart, liver, spleen, lung and kidney

The expression levels of Ki-67 and p-JAK2 in tumor tissues were detected by immunohistochemical analysis. The results showed that compared with the negative control group, the positive rates of Ki-67 and p-JAK2 in the 400 mg/kg and 800 mg/kg PSP treatment groups were significantly reduced (*p < 0.05*). Conversely, the Tunel staining increased in the PSP-treated group, indicating that PSP induced apoptosis of tumor cells (*p* < 0.05) (Fig. 9A). Meanwhile, western blotting results confirmed that the expression levels of p-JAK2 and p-STAT3 in the 400 mg/kg and 800 mg/kg PSP treatment groups were significantly reduced compared with the control group (Fig. 9B). These data suggest that PSP inhibits tumor proliferation and induces apoptosis by inhibiting the JAK2-STAT3 signaling pathway. HE staining showed that PSP treatment caused no significant damage to the major organs of the mice, including heart, liver, spleen, lungs, and kidneys. Also, no inflammatory cells were observed, which indicated that PSP had no obvious toxicity and an excellent safety in vivo (Fig. 10).

## Discussion

Studies have confirmed that PSP is a widely used, safe and non-toxic active ingredient of traditional Chinese medicine with great potential due to its pharmacological activities such as enhancing body immunity, anti-tumor, protecting liver and lowering blood lipids [9]. Previous studies on the anti-tumor mechanism of PSP were mostly applied to leukemia, gastric cancer, etc., but there were few studies on the role of PSP in breast cancer. This study used network pharmacology and experimental validation to explore the role and mechanism of PSP in breast cancer.

Network pharmacology, as an effective means to systematically integrate drugs, targets and diseases, provides a new way to study the mechanism of action of traditional Chinese medicine [10]. In this study, 287 targets of PSP and 183 targets of breast cancer were obtained by network pharmacology. A total of 10 targets at the intersection of PSP and breast cancer were selected to draw Venn diagram and construct PPI network. Then GO and KEGG enrichment analysis was performed for these 10 target genes. From the results of GO enrichment analysis, it can be found that PSP may enhance human immunity by enhancing the response to hormones and regulating enzymatic activity. It may inhibit the growth of malignant tumors by regulating apoptosis signals, and may also participate in the reversal of drug resistance of some chemotherapy or targeted drugs. This is basically consistent with the mechanism of PSP mentioned in previous studies. KEGG enrichment analysis showed that PSP was involved in the regulation of PI3K-AKT, HIF-1, JAK-STAT and other cancer-related signaling pathways.

Molecular docking is a computer-based approach widely used in drug discovery. Docking enables the identification of novel compounds of therapeutic significance and the prediction of ligand-target interactions at the molecular level [11]. Molecular docking results indicated that the binding energy values between PSP and the target JAK2, PARP1, AR and HPGDS were all lower than − 6.5 kcal/mol, indicating that they had good binding activity and could form a stable binding conformation. Among them, JAK2 has the highest binding activity and is the focus of our attention.

Janus kinase 2(JAK2), a member of the Janus enzyme family, is a non-receptor tyrosine kinase that is essential for signal transduction of various cytokine receptors [12]. Previous studies have shown that JAK2 was overactive in triple-negative and HER-2 positive breast cancers [13, 14]. Therefore, JAK2 inhibitors have the potential to become a new therapeutic method for triple-negative and HER-2 positive breast cancers [15]. As a substrate of JAK2, activated signal transduction and transcription factor 3(STAT3)can bind to its target gene promoter region and up-regulate the expression of genes involved in cell cycle progression, proliferation, anti-apoptosis, angiogenesis and metastasis [16, 17].The JAK2-STAT3 pathway has been shown to play an important role in the proliferation and apoptosis of various solid cancers and hematologic malignancies [18–21]. In particular, the constitutive active form of STAT3 has been detected in more than 50% of breast cancers [22], suggesting that the JAK2-STAT3 pathway plays an important role in breast cancer tumorigenesis. Studies have shown that the JAK2-STAT3 signaling pathway was involved in the growth of CD44^+^/CD24^−^ stem-like cell populations in human breast cancer cells [23]. SLSI-1216, a small molecule STAT3 inhibitor, inhibits the proliferation and tumor growth of triple-negative breast cancer cells by inducing apoptosis [24]. Methylselenic acid inhibits breast cancer tumor growth by inhibiting JAK2/STAT3 pathway [25]. Therefore, targeting JAK2-STAT3 signaling is considered a promising strategy for the treatment of breast cancers. In this study, combined with the results of network pharmacology and molecular docking, we speculate that the mechanism of PSP therapy for breast cancers may be related to the promotion of apoptosis and inhibition of tumor growth by targeting JAK2-STAT3 signaling pathway. Therefore, we conducted experiments to verify the possibility of target screening results.

We used different breast cancer cell lines for experimental validation. It is important to note that before starting the experiments, we conducted pre-experiments to determine the optimal concentration for PSP. The results showed that PSP had no inhibitory effect on breast cancer cells and even promoted the proliferation of breast cancer cells when PSP concentration was less than 2 mg/ml. We think this is hormesis. That is, a certain compound can inhibit the growth of organisms at high concentrations, but organisms can neutralize or resist the inhibitory effect of the compound at low concentrations, that is, a self-correction of organisms [26]. Therefore, subsequent cell experiments were carried out with PSP concentration of 2.5 mg/ml, 5 mg/ml and 10 mg/ml. The results showed that PSP can significantly inhibit the proliferation and induce apoptosis of MDA-MB-231, MCF-7 and SUM-159 human breast cancer cell lines in a concentration-dependent manner. Western blotting results showed that the expression levels of p-JAK2 and p-STAT3 in breast cancer cells were significantly decreased after PSP treatment.

Finally, we used MDA-MB-231 tumor bearing mice to investigate the role of PSP on tumor growth in vivo, which provides a scientific rationale for the treatment of breast cancer patients. Our results showed that PSP significantly reduced tumor volume in mouse models. In addition, immunohistochemical results of tumor tissues provided strong molecular evidence to support the prediction of network pharmacology that PSP can mediate tumor growth by inhibiting the JAK2-STAT3 signaling pathway.

In summary, PSP can inhibit cell proliferation and induce cell apoptosis in breast cancer cells, and the mechanism of its treatment for breast cancer is mainly to inhibit JAK2-STAT3 pathway. This finding has been confirmed both in vivo and in vitro. At the same time, we successfully predicted the potential target of PSP treatment of breast cancer based on network pharmacology combined with molecular docking, providing a reasonable direction for future breast cancer treatment and drug development research.

## Data Availability

The data and material used to support the findings of this study are available from the corresponding author upon request.
